# Adult ADHD and comorbid disorders: clinical implications of a dimensional approach

**DOI:** 10.1186/s12888-017-1463-3

**Published:** 2017-08-22

**Authors:** Martin A. Katzman, Timothy S. Bilkey, Pratap R. Chokka, Angelo Fallu, Larry J Klassen

**Affiliations:** 1 START Clinic for Mood and Anxiety Disorders, 32 Park Road, Toronto, ON M4W 2N4 Canada; 2 Adler Graduate Professional School, Toronto, ON Canada; 3 Northern Ontario School of Medicine, Laurentian and Lakehead University, Thunder Bay, ON Canada; 4 Department of Psychology, Lakehead University, 955 Oliver Road, Thunder Bay, ON P7B 5E1 Canada; 5 Ontario Bilkey ADHD Clinics, 400 Bayfield Street, Suite 245, Barrie, ON L4M 5A1 Canada; 6 Chokka Center for Integrative Health, 2603 Hewes Way NW #201, Edmonton, Alberta T6L 6W6 Canada; 7 University of Alberta, 1E1 Walter Mackenzie Centre, Edmonton, AB T6G 2R7 Canada; 8 Clinique Woodward, 717 rue Woodward, DIEX Research Inc., Sherbrooke, Quebec, J1G 1W4 Canada; 9 Eden Mental Health Centre, 1500 Pembina Ave, Winkler, MB R6W 1T4 Canada

**Keywords:** Adult ADHD, Neurobiology, Psychiatric comorbidity

## Abstract

Attention-deficit/hyperactivity disorder (ADHD) in the adult population is frequently associated with comorbid psychiatric diseases that complicate its recognition, diagnosis and management.

The prevalence of ADHD in the general adult population is 2.5% and it is associated with substantial personal and individual burden. The most frequent comorbid psychopathologies include mood and anxiety disorders, substance use disorders, and personality disorders. There are strong familial links and neurobiological similarities between ADHD and the various associated psychiatric comorbidities. The overlapping symptoms between ADHD and comorbid psychopathologies represent challenges for diagnosis and treatment. Guidelines recommend that when ADHD coexists with other psychopathologies in adults, the most impairing condition should generally be treated first.

Early recognition and treatment of ADHD and its comorbidities has the potential to change the trajectory of psychiatric morbidity later in life. The use of validated assessment scales and high-yield clinical questions can help identify adults with ADHD who could potentially benefit from evidence-based management strategies.

## Background

Attention-deficit/hyperactivity disorder (ADHD) is a psychiatric disorder associated with considerable personal and societal burden. While ADHD is well recognized in the pediatric population, where it was first described as a clinical diagnosis in the 1930s [1], focus has shifted to include the recognition and management of the condition in adults [2]. Often, adult ADHD has a more heterogeneous clinical presentation that transcends the typical motor symptoms described in pediatric populations, and includes a wider spectrum of emotional dysregulation and functional impairment.

As our diagnostic systems are evolving towards a more dimensional approach to the classification of mental disorders, so too is our understanding of adult ADHD [3]. Today, practicing clinicians recognize the heritability of ADHD and the wide variability in clinical presentation of adult ADHD. As many as 80% of adults with ADHD have at least one coexisting psychiatric disorder [4, 5], including mood and anxiety disorders, substance use disorders (SUD), and personality disorders. This can complicate the recognition and diagnosis of ADHD in adults, and despite ongoing clinical controversy, the bulk of evidence suggests that ADHD remains under-recognized and under-treated in the adult population [6]. Despite the challenges of recognizing ADHD in adults with complex clinical presentations, there are effective treatments available that have been demonstrated to improve clinical and functional outcomes, including important elements of psychosocial functioning such as social relationships, workplace performance, and parenting skills. This makes the recognition and successful management of ADHD in adults extremely gratifying for clinicians. It has been suggested that early and optimal treatment of ADHD could potentially alter the trajectory of psychiatric morbidity down the road by preventing the emergence of psychiatric comorbidities such as mood and anxiety disorders or SUDs [7, 8].

Thus, the goals of this article are to review the available evidence on the prevalence, burden, and neurobiology of adult ADHD, to describe how a practical, dimensional approach can help clinicians identify ADHD in patients with complex presentations, and to inform appropriate management decisions in order to improve patient outcomes in this under-treated population. With the recognition that the evidence base continues to evolve and that there is a lack of quality evidence to guide the management of complex patient presentations, this article answers some common clinical questions based on available evidence as well as our collective experience in the management of adult ADHD and comorbidities.

## Main text

### Methods

PubMed was searched for articles in English published between 1996 and 2016, using the following search terms: “adult ADHD” in combination with anxiety; addiction; affective dysregulation; alcohol; bipolar; burden; catecholamine deficit; cocaine; cognition; depression; dimension; dimensional; disability; executive functioning; functioning; guideline; heritability; life expectancy; mania; marijuana; mortality; neurobiology; nicotine; personality disorders; prevalence; prevention; recommendation; risk factors; and substance use. Clinical trials, guidelines, meta-analyses, and systematic reviews were selected by the authors for inclusion in this review.

In total, 113 unique articles were identified; 8 were excluded at the title filtering stage, 7 at the abstract filtering stage, and another 7 after a full-text review. Overall, 22 articles were excluded and 91 were included. Exclusion criteria included: articles about disorders other than ADHD (such as Parkinson’s, phenylketonuria, brain injury); studies of pediatric or adolescent populations; and studies involving alternative therapies rather than psychological therapy or pharmacotherapy. Manual searches of the reference lists of identified articles and other interesting published works including authoritative texts were also selected, bringing the total number of articles included in this review to 150.

### Prevalence of ADHD

ADHD has an estimated childhood prevalence of 4% to 7% [9] with increasing evidence pointing to its continuation into adulthood for between 15% and 65% of individuals [10]. Recent evidence supports the view that children with ADHD do not “outgrow” the disorder when they reach adulthood, and furthermore, that adult ADHD is not necessarily a continuation of childhood ADHD since a substantial proportion of adults with ADHD lack a history of the disorder in childhood [11–13]. Taken together, such observations suggest the existence of two separate syndromes that have distinct developmental trajectories [11].

The general population prevalence of ADHD in adults has been estimated to be 2.5% (95% confidence interval [CI] 2.1–3.1) [14], with adults with ADHD presenting with symptoms such as: failing to pay attention to detail, difficulty organizing tasks and activities, excessive talking or fidgeting, difficulty relaxing, overworking, forgetfulness, and distractibility [15–17]. Nonetheless, despite the relatively high prevalence of ADHD in adults, it is often unrecognized in patients who present to the clinic (reviewed by Ginsberg et al., 2014) [18]. This is particularly true for females, who are a largely unrecognized population for several reasons. Notably, childhood ADHD is usually diagnosed after a referral from parents or teachers, with boys being more likely to be referred for treatment since they present primarily with external symptoms such as hyperactivity, which are inevitably more noticeable to others [19]. Conversely, females with ADHD are more likely to have internalizing symptoms, resulting in a later diagnosis, and greater time for developing strategies to mask core symptoms [20]. Despite this, one meta-analysis reported that females with ADHD often have greater intellectual impairments than males with the disorder [19], highlighting the importance of recognizing and appropriately managing this under-represented population.

### Burden of adult ADHD

Adult ADHD is associated with profound functional and psychosocial disability, leading to serious personal and societal costs. Its most prominent feature is attentional dysfunction, associated especially with impairment in focused and sustained attention [21]. Individuals with ADHD also experience neuropsychological difficulties associated with deficient inhibition [22], memory [22], executive functioning [23, 24], decision making [25], and emotional dysregulation [26]. Adult ADHD can have negative consequences for individuals’ self-esteem and the quality of interpersonal relationships, with both colleagues and significant others [27, 28]. For example, in a community sample of 1001 adults, those with ADHD were significantly more likely to have been divorced (28% vs 15% controls, *P* ≤ 0.001) and were significantly less satisfied with their personal, social and professional lives [29]. ADHD is associated with educational difficulties, requiring extra help, attending special classes, repeating grades [30], as well as higher rates of academic suspension and drop outs [31]. College students with ADHD have reduced grade point averages and are less likely to graduate than students without ADHD [32]. Later in life, adults with ADHD experience challenges with time management, organization, and self-regulation, which can result in employment and financial problem [27, 33]. One study estimated the individual income reduction in adults with ADHD in the United States to be between $8900 and $15,400 annually [34].

The detrimental effects of ADHD on overall health and safety provide additional imperative to appropriately recognize and manage this debilitating disorder. Adult ADHD has been associated with poorer driving and a higher incidence of traffic citations and motor vehicle accidents [35]. A recent study found that Japanese adults with ADHD visited physicians 10 times more often than a non-ADHD control group, and had rates of emergency room visits and hospitalization three times greater than controls [28]. Individuals with ADHD in Denmark have a lower life expectancy and more than double the risk of death than adults without ADHD [36]. This was mostly attributed to accidental death and characteristics associated with ADHD such as risk-taking behaviour.

In addition to its substantial burden at the individual level, adult ADHD is often associated with considerable societal costs. Notably, there have been consistent associations between adult ADHD and unemployment [34, 37]. In one study, adults with ADHD were 42% less likely to be employed full-time as were adults without ADHD (rates of full-time employment: 34% vs 59%, respectively, *P* < 0.001) [34]. The associated loss of workforce productivity has been estimated to cost $67 to $116 billion annually in the United States alone [34]. A more recent study estimated the overall annual cost of ADHD in the United States at between $143 and $266 billion, largely due to productivity and income losses [38]. ADHD has been associated with increased criminality [39], with one study reporting that 47% of patients with ADHD had at least one criminal sentence [4]. Another study estimated the prevalence of ADHD among long-term inmates of a prison at 40% [40].

Along with these functional and psychosocial impairments, ADHD is associated with a higher risk of developing mood and anxiety disorders. In many studies, ADHD has been associated with comorbid depression, anxiety disorders, bipolar disorder, and substance use disorder [27, 30, 40–42]. The National Comorbidity Survey reported that adults with ADHD are three times more likely to develop major depressive disorder (MDD), six times more likely to develop dysthymia, and more than four times more likely to have any mood disorder [37]. Most notably, individuals with ADHD are twice as likely to experience substance abuse or dependence [43]. These comorbidities present important clinical challenges since their co-occurrence results in greater disease burden and more severe illness courses than ADHD or mood and anxiety disorders alone [44].

### Neurobiological and genetic concepts

There are strong familial links between ADHD and psychiatric comorbidities such as bipolar disorder, suggesting a genetic contribution [17]. One possible explanation is that ADHD and mood disorders stem from similar neurobiology. Recent studies have demonstrated that similar regions of the brain are involved in ADHD and psychiatric disorders [17]. Neuroimaging studies have implicated differences in volume and activity in the frontal lobe, which is responsible for attention, behaviour selection, and emotion [16]. Studies of neurotransmitters have also pointed to abnormalities in dopamine (DA) and norepinephrine (NE) signaling [16, 45], thus corroborating Volkow et al.’s (2012) conclusion that methylphenidate-elicited dopamine increases were associated with therapeutic response in individuals with ADHD [46].

Interestingly, the main neural pathway that modulates emotional affect comprises the limbic-cortical-striatal-pallidal-thalamic (LCSPT) circuits, consisting of connections between the orbital and medial prefrontal cortex (OMPFC), ventromedial striatum, ventral pallidum, hippocampal subiculum, mediodorsal and midline thalamic nuclei, and amygdala [47]. These circuits integrate higher cognitive functions with visceral information and external environmental conditions to affect mood and emotional states, through reciprocal connections with regions of the cortex that are involved with control of higher cognitive functions as well as regions associated with regulation of autonomic functions, including the periaqueductal gray and the hypothalamus [48]. While the neuronal activity within LCSPT circuits is predominantly glutamatergic and is locally modulated through the gamma-aminobutyric acid (GABA) system [49], the activity of the LCSPT circuit with its related organs can be modulated by a variety of other neuromodulators, including the endocannabinoids [50] and the various monoamines. In fact, dysfunction in LCSPT circuits and its related modulating neurotransmitter systems has been implicated as playing a key role in MDD [47]. In part, this may be related to deficits in reward processing with altered monoamine signalling having been implicated as the underlying mechanism of this effect. This deficit in reward processing characterized as low hedonic tone [51, 52], is hypothesized to be at least in part related to deficits in modulation of this circuitry [53]. These deficits, which result in altered sensitivity to reinforcement, have been shown in children with ADHD who have been reported to preferentially respond to immediate rewards, but not when the rewards are delayed and therefore only exhibit conditioning to immediate rewards [54], which parallels some of the anomalous changes in the neural pathways that regulate reward and motivation [55], matching those in depression.

### Diagnostic challenges

As such, one can imagine this low hedonic tone as a key feature shared by MDD and resulting from a shared dysfunction in monoamine signaling, particularly in the ventral striatum [52]. In support, abnormalities in DA and NE signalling have been reported in both MDD and ADHD, suggesting a potentially shared underlying pathophysiology, at least in some individuals [56–59]. Interestingly, treatment with methylphenidate normalizes the hypoperfusion of prefrontal areas and is associated with corresponding improvement in ADHD symptoms [60–65]. Perhaps this explains the findings of Sternat et al. (2016) who reported that 34% of patients referred for treatment resistant depression (TRD) met criteria for ADHD with predictors of this comorbidity including selective serotonin reuptake inhibitor (SSRI) failure and chronic anhedonia [52].

Overlapping symptomology between ADHD and mood, anxiety, or SUDs present several barriers to diagnosis and treatment. Studies have concluded that emotional dysregulation is a distinctive attribute of adult ADHD psychopathology, however these symptoms may be misdiagnosed as a mood disorder [66–68]. Similarly, ADHD symptoms may be masked by substance use [43]. Physicians are often more familiar with mood and anxiety disorders, which may contribute to misdiagnosis and delays in treating ADHD in adults [69]. It has been suggested that stress, depression, and anxiety could manifest as a consequence of undiagnosed and untreated ADHD [70]. The result is that many individuals with ADHD receive treatment for comorbid mood disorders, but not for ADHD [37, 71, 72]. Overall, these challenges have contributed to an under-diagnosis and under-treatment of adult ADHD [18]. According to a National Comorbidity Survey Replication in the United States (*n* = 3199), only 11% of adults with ADHD were receiving treatment [37]. In part, these situations are exacerbated by the development of mistaken beliefs regarding the over-diagnosis and over-treatment of ADHD [73], which further lower the likelihood of patients receiving the diagnosis and targeted treatment to change their life trajectory.

### The Spectrum of ADHD and other psychopathologies

**Fig. 1:**
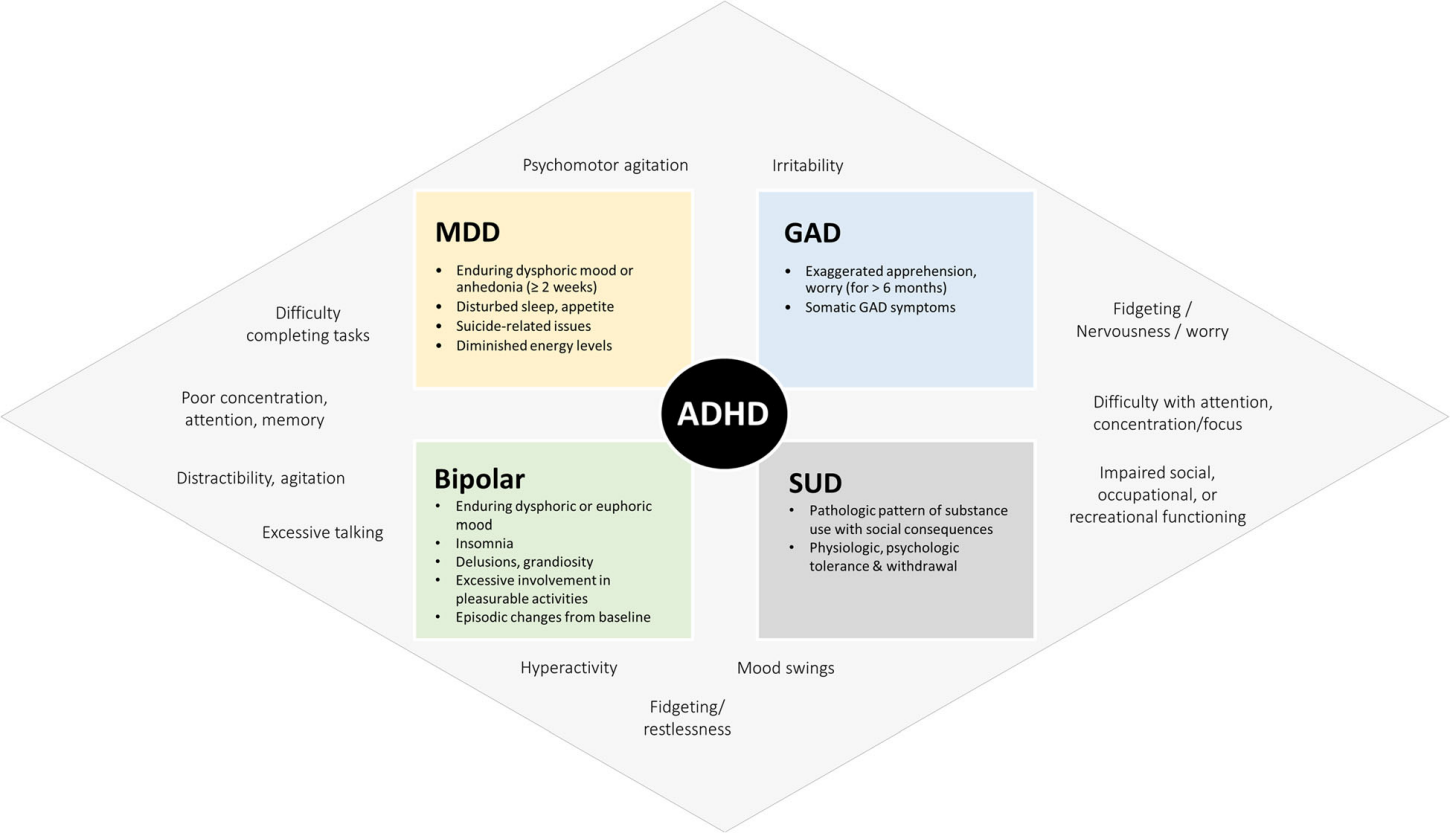
Overlapping and distinctive features of ADHD and common psychiatric comorbidities (compiled from: Searight et al., 2000 [149]; Culpepper and Mattingly, 2008 [150]; Klassen et al., 2010 [17]; Bond et al., 2012 [16]; Mancini et al., 1999 [85]; CADDRA, 2011 [107]; Mao and Findling, 2014) [84]

The most common psychiatric comorbidities that co-occur with ADHD in adults are depression, anxiety disorders, bipolar disorder, SUDs and personality disorders. The overlapping and distinctive features of these disorders are summarized in Fig. 1. Given the considerable overlap between these disorders, the conceptualization of ADHD as a spectrum using a dimensional rather than a categorical approach to diagnosis and treatment has been proposed [3]. This framework is in keeping with work in other areas of psychiatry, notably the National Institute of Mental Health’s Research Domain Criteria (RDoC) initiative and the most recent Diagnostic and Statistical Manual of Mental Disorders 5th Edition [15], which both espouse dimensional approaches to the diagnosis and classification of mental disorders as a strategy to facilitate mental health research [74]. An important driver of this paradigm shift towards dimensional approaches to mental health research is the effort “to better understand basic dimensions of functioning underlying the full range of human behaviour from normal to abnormal” [75].

- *Bipolar Disorder*

ADHD has a high prevalence of comorbidity with bipolar disorder. Rates of ADHD comorbidity in bipolar disorder have been estimated between 9.5% and 21.2%, and rates of comorbid bipolar disorder in ADHD at 5.1% and 47.1% [76]. Bipolar I disorder is more common in individuals with comorbid ADHD than is bipolar II disorder [76]. Characteristics of the manic or elevated phase of bipolar disorder that overlap with ADHD include restlessness, talkativeness, distractibility, and fidgeting [17]. The distinctive features of bipolar disorder, largely characterized by the depressive phase, as well as the episodic course of symptoms, can help to elucidate a differential diagnosis [17]. Several studies have suggested that comorbid ADHD hastens an earlier age of onset of bipolar disorder. In one study, 65% individuals with ADHD had early onset of bipolar disorder (at under 18 years of age), compared with only 20% of individuals who did not have comorbid ADHD [77]. Other studies have reported that bipolar patients with comorbid ADHD experienced an earlier average age of mood disorder onset by 5 years [78] or 6 years [44] compared to individuals without ADHD. In addition to an earlier age of onset, bipolar individuals with comorbid ADHD have demonstrated worse overall course of illness with shorter periods of wellness, more frequent episodes of mania and depression, and higher instance of additional comorbid psychiatric conditions including anxiety and substance use disorders [78]. Questions regarding the role of stimulants in bipolar depression remain unresolved [79, 80]. This might be related to raised hedonic tone in bipolar depression [51]. Furthermore, the use of long-acting stimulants in individuals with ADHD and bipolar disorder has been advocated by some once mood has been stabilized with an appropriate mood stabilizer [17]. Still, concerns remain in regards to the potentially increased risk of stimulant-associated mania/hypomania in bipolar disorder patients [81].

b) *Dysthymia/Depression*

ADHD and dysthymia/depression co-occur frequently, with studies reporting prevalence rates of depression in individuals with ADHD ranging from 18.6% [37] to 53.3% [4]. Similarly, studies have reported comorbid ADHD in individuals with depression at rates of 9% to 16% [69], with a mean rate of 7.8% [16]. Individuals with comorbid ADHD and depression have a high disease burden including lower self-reported quality of life than those with MDD alone [44]. One important consideration is the possibility of depressive symptoms manifesting as a result of coping with lower hedonic tone in ADHD [51] rather than being representative of a depressive disorder separate from ADHD [69]. A recent study reported that 28% of individuals referred to a tertiary clinic for mood and anxiety assessment had undetected ADHD [82]. Factors that were significantly predictive of undetected ADHD included the number of SSRIs previously received (*P* < 0.03). This is not surprising, since serotonergic agents alone would not be expected to improve ADHD symptoms, which typically respond to catecholaminergic agents such as noradrenalin-dopamine reuptake inhibitors or psychostimulants. Purely serotonergic activity lowers dopamine and norepinephrine levels via effects on 5-HT_2C_ and 5-HT_2A_ interneurons, respectively [83]. As well, this presentation may represent demoralization as a result of ADHD and subsequent emergence of symptoms such as anhedonia, sleep issues, and irritability. Therefore, the key to successfully diagnosing concurrent MDD is facilitated by recognizing the presence of a static depressed affect, appetite changes, or suicidal ideation [69, 84].

c) *Anxiety Disorders*

The risk for anxiety disorders is higher in individuals with ADHD than in the general population [85, 86] with rates approaching 50% [37]. Comorbid ADHD is more common in individuals with a primary diagnosis of social phobia than panic disorder [85]. Individuals with anxiety disorders who have comorbid ADHD tend to have more severe anxiety symptoms, earlier age of onset of anxiety, and more frequent additional comorbid psychiatric diagnoses and substance use than those who do not have ADHD [85]. ADHD is often diagnosed later in individuals who have comorbid anxiety than in those without anxiety, possibly because the presence of anxiety may inhibit impulsivity [87]. Nonetheless, one might also understand the comorbidity between the anxiety disorders and ADHD as being related to common neurobiological deficits associated with poor prefrontal activity and deficits in top-down regulation. Neuroanatomical gradations in “cool” processing appear to be related to prefrontal dysfunction involving the dorsolateral prefrontal cortex (dlPFC), and the parietal cortex, insula cortex and anterior cingulate cortex (ACC) are also critically involved in executive function as part of a frontal-parietal executive control network [88–93]. Support for this model is derived from anatomical findings in children with ADHD showing delayed maturation in terms of the thickness of the entire cortex, with the greatest delays in prefrontal cortex (PFC) and ACC [94].

d) *Substance Use Disorder*

Possibly the most common comorbid condition with ADHD is SUD, particularly alcohol and/or nicotine, cannabis and cocaine use [95]. Substance abuse or dependency is approximately twice as common in individuals with ADHD as it is in the general population [43]. There is a particularly strong association between ADHD and cigarette use, with these populations demonstrating stronger physical dependence to nicotine when compared to individuals without ADHD [96]. The association between ADHD and SUD is bidirectional, and stems from various sources including neurobiological factors, other comorbid psychiatric disorders, behavioural characteristics such as novelty-seeking or impulsivity, and attempts to self-medicate ADHD symptoms [43]. In support of the latter, individuals with ADHD more frequently report the use of substances in order to manage their mood or as sleep aids [97].

The burden of comorbid ADHD with SUD is substantial. Studies have found that ADHD in individuals with SUD is associated with earlier onset of substance use, increased likelihood of suicide attempts, more hospitalizations, higher rates of poly-substance abuse, less likelihood of achieving abstinence, and lower rates of treatment adherence [43, 98, 99]. Overall, the co-occurrence of ADHD and SUD can result in a more severe course of both substance use and psychiatric symptoms and outcomes. It is therefore important to screen for ADHD in patients presenting with SUD and vice-versa [41].

Although a link between treatment of ADHD with psychostimulants and later development of SUD has been proposed, this is not supported by the literature [95]. Notably, evidence suggests that the use of methylphenidate in children may in fact reduce substance use and abuse in adolescence and adulthood by up to 85% (reviewed by Klassen et al., 2012) [95].

e) *Personality Disorders*

The literature on comorbid ADHD and personality disorders is sparse compared to other psychiatric comorbidities. Reports suggest that personality disorders are present in more than 50% of adults with ADHD, most commonly cluster B and C personality disorders, and 25% of individuals have two or more personality disorders [100]. Importantly, individuals with ADHD and personality disorders have more severe impairment [100], exhibit lower response rates to methylphenidate treatment compared to adults with ADHD alone [101], and have poorer persistence on ADHD therapy [102]. Adults with ADHD and predominant features of emotional dysregulation have a higher incidence of comorbid personality disorders (74%) than do adults with ADHD with predominantly inattentive features (32%) [103], lending further credence to the conceptualization of ADHD as a dimensional disorder that occurs along a spectrum of clinical presentations and severity.

ADHD and personality disorders often co-occur with other axis I disorders. For instance, 18% of adults with ADHD and depression and 23% of adults with ADHD and bipolar disorder are estimated to also have a personality disorder [44]. The substantial burden of comorbid ADHD and personality disorders is underscored by the high co-occurrence of these conditions in incarcerated individuals. In one study, a staggering 96% of imprisoned adults with ADHD had a lifetime history of antisocial personality disorder [40]. Other types of personality disorders were also over-represented in this population, including borderline (74%), paranoid (74%), narcissistic (65%), obsessive-compulsive (52%), passive-aggressive (48%), and avoidant (48%).

There is a paucity of clinical trials evaluating management strategies for personality disorders in general [104], let alone with comorbid ADHD. It is plausible that in some individuals, adult ADHD may manifest as a personality disorder and that targeting treatment to ameliorate symptoms of ADHD might also improve features of antisocial behaviour.

### Distinguishing ADHD from common psychiatric comorbidities

It is important to determine if a patient presenting with one of the above-listed psychiatric disorders also has comorbid ADHD. This may be difficult, in part because of the difficulties associated with establishing a correct diagnosis. According to the DSM-IV-TR, ADHD is defined on the basis of the diagnosis being made before age 7 [105], but the DSM-5 extended this to age 12 [15]. Nonetheless, some individuals will be able to overcome their deficits such that diagnosis will be delayed. Compensating skills may delay or prevent diagnosis if one follows the strict DSM definition. In fact, as noted by Moffitt et al. (2015) [12], when one looks at individuals with early onset (i.e. diagnosed before age 12), they exhibit fewer university degrees, lower intelligence quotients (IQs) by almost one standard deviation, more requirements for government support, more disability, more criminal convictions, more childhood depression and more conduct disorder. Thus, those with delayed onset of symptoms may in fact be able to compensate thereby delaying diagnosis, but they may eventually require treatment nonetheless. This compensation and delayed diagnosis may contribute to unrecognized ADHD, which has been associated with poor treatment response or noncompliance due to forgetfulness, or perceived lack of improvement of symptoms [66], or mismanagement where the medication will only address the problems it is designed to target (e.g. SSRIs will not address the primary premorbid ADHD contributing to the trajectory of depression, bipolarity, anxiety and substance abuse problems). In fact, treating ADHD has been shown to prevent worsening comorbidities with depression, bipolarity, anxiety and substance use disorders [7, 8].

Thus, we propose three key questions that clinicians can ask in order to help identify red flags suggestive of an ADHD diagnosis in complicated patients:

- Have you had long-standing and consistent problems with attention and distractibility?
- Have your current complaints been present over the last 10 or 20 years?
- If I could see you in the classroom when you were a child, what would you be like?

If these questions suggest a possible positive ADHD diagnosis, an in-depth clinical interview should be undertaken using a screening instrument such as the Adult ADHD Self-Report Scale (ASRS), the Wender-Reimherr Adult Attention-Deficit Disorder Scale (WRAADDS), or the Conners Adult ADHD Rating Scales (CAARS). Another tool for assisting diagnosis is FAST MINDS (Forgetful; Achieving below potential; Stuck in a rut; Time challenged; Motivationally challenged; Impulsive; Novelty seeking; Distractible; Scattered) [106]. Once a positive diagnosis of ADHD has been established, the severity of functional impairment and quality of life can be assessed using the Weiss Functional Impairment Rating Scale (WFIRS) and the Adult ADHD Quality of Life Scale (AAQoL) [84].

### Treatment considerations

Several published articles have presented reviews and recommendations concerning treatment options and algorithms [16, 107]. Treatment selection must be informed first and foremost by efficacy in terms of functional outcomes. Functional outcomes include symptom reduction, but also extend to improved daily functioning and increased quality of life [108]. Indicators of improved functioning include more efficient at working or studying, more stable relationships, success in containing aggressive impulses, and improved parenting [72]. Long-term efficacy as well as adherence to treatment is also crucial to success.

#### Pharmacologic treatments

Pharmacologic treatments for ADHD are usually divided into stimulants and non-stimulants. Stimulant medications include methylphenidate, mixed amphetamine salts, and lisdexamfetamine dimesylate. Non-stimulants used in ADHD treatment include atomoxetine and alpha-2-adrenergic agonists. Antidepressants such as venlafaxine and bupropion have also been evaluated as treatment options for ADHD, with some evidence of benefit in addressing ADHD symptoms [16, 109]. One systematic review and meta-analysis of treatments for ADHD concluded that immediate-release methylphenidate was superior to other treatments in terms of benefits and harms [110]. It also supported the efficacy of atomoxetine, long-acting bupropion, and extended-release stimulants, but found that short-acting stimulants had similar risk-profiles to these other options, with greater efficacy in terms of symptom reduction. Recommendations for the pharmacologic management of adult ADHD are described in Table 1.

**Table 1:** Summary of Canadian ADHD Resource Alliance (CADDRA) guidelines for medical treatment of adults with ADHD [107]

Line of therapy	Recommended treatment(s)
First-line	Long-acting stimulants Amphetamine mixed salts (Adderall XR) Lisdexamfetamine dimesylate (Vyvanse) Methylphenidate HCl (Biphentin, Concerta)
Second-line/adjunctive	Long-acting non-stimulants Atomoxetine (Strattera)
Second-line/adjunctive	Short- or intermediate-acting stimulants Dextro-amphetamine sulphate (Dexedrine, Dexedrine Spansule) Methylphenidate HCl (Ritalin, Ritalin SR)

*HCl* hydrochloride, *XR* extended release, *SR* sustained release

Another important treatment consideration is the potential for the effective treatment of ADHD to improve functional outcomes of patients with comorbid conditions. Many studies have reported improvements in comorbid psychiatric symptoms when ADHD is effectively treated. For instance, atomoxetine has been associated with improvements in both ADHD and comorbid anxiety [111] and depressive [112] symptoms. Other studies have demonstrated the efficacy of co-administration of SSRIs or serotonin-norepinephrine reuptake inhibitors (SNRIs) with stimulants on functional outcomes in ADHD with comorbid anxiety or depressive symptoms [113, 114].

Perhaps more exciting is the concept that early and optimal treatment of ADHD could potentially prevent the later development of psychiatric comorbidities. In a 10-year longitudinal follow-up study of male youths with ADHD, Biederman et al. (2009) [7] found that those who were treated with stimulants had a significantly lower risk of developing comorbid depressive and anxiety disorders as adults, and were also significantly less likely to have impaired functional outcomes, than those who were not treated. Similarly, adolescents with ADHD treated with stimulants were found to have a significantly lower risk of cigarette smoking and subsequent development of SUD after 5 years of follow-up [115]. Taken together, these observations suggest that pharmacologic therapy for ADHD in young adults could change the trajectory of psychiatric morbidity in adulthood. Such findings provide powerful support for the early and aggressive treatment of ADHD.

A final important treatment consideration is safety and tolerability. Both stimulant and non-stimulant medications have possible side effects, which must be taken into account. Common side effects of stimulants include headache, appetite suppression, nausea, dry mouth, mood fluctuations, difficulty sleeping, jitteriness, increased heart rate, and increased blood pressure [116]. Generally the severity and risk of these side effects is considered minimal. However, due to the possibility of serious cardiac adverse events, it is recommended that patients be screened for both family and personal histories of cardiac conditions prior to prescription of stimulant medications [107]. Side effects vary depending on the type of non-stimulant employed, but common side effects of atomoxetine include appetite suppression, dry mouth, insomnia, constipation, vomiting, dizziness, fatigue, nausea, dyspepsia and mood swings [109]. However, most experts agree that minimal lab investigations are needed prior to the initiation of ADHD medications in adults, particularly the psychostimulants. For instance, routine bloodwork is not necessary in most individuals, and only at-risk individuals may require monitoring of blood pressure, heart rate, and electrocardiogram prior to starting psychostimulants.

#### Non-pharmacological treatments

Non-pharmacological interventions play a central role in the management of ADHD. Evidence supports the superiority of multimodal approaches utilizing pharmacotherapy and psychosocial and/or behavioural interventions to target the core symptoms of ADHD and for the improvement of functional outcomes [107, 117]. Similarly, the addition of psychotherapeutic approaches to pharmacotherapy in adults with ADHD whose symptoms persist despite medication has been shown to improve symptoms and functioning [118]. Notably, recent research suggests that cognitive behavioural therapy (CBT) has bidirectional efficacy for both ADHD and depressive disorders [119]. Table 2 lists non-pharmacological strategies recommended by the CADDRA guidelines for ADHD.

**Table 2:** Summary of CADDRA recommendations for non-pharmacological treatments for ADHD [107]

Psychosocial intervention or treatment	Key components
Psychoeducation	• Strategy instruction (e.g. sleep management, anger control) • Self-talk development • Organizational skills development • Information on ADHD diagnosis, assessment, investigations, treatments, myths • Community resources and support groups
Behavioural interventions	• Rewards and consequences (e.g. response cost, point systems, token economies) • Environmental management • ADHD coaching • Lifestyle change (e.g. diet, exercise, sleep)
Social interventions	• Social skills training • Anger management • Supervised recreation • Parenting skills training
Psychotherapy	• Self-talk strategies • Cognitive behavioural therapy (CBT) • Interpersonal therapy • Family therapy • Expressive arts therapy • Supportive counseling
Educational / vocational accommodations	• Academic remediation • Specialized academic or workplace interventions

Psychotherapeutic modalities are also central to the management of the most common psychiatric comorbidities in adults with ADHD, namely SUD, depression, anxiety, and bipolar disorder. However, prospective studies of psychosocial strategies in comorbid ADHD populations are scarce. One study in 419 outpatients with ADHD, of which 38% had at least one comorbid axis I psychiatric disorder, showed that multimodal strategies are more effective than psychosocial or pharmacotherapeutic strategies alone for improving symptoms of ADHD, but the effects on depressive symptoms were not statistically significant [117]. A small study of 54 adults with ADHD reported that the addition of psychotherapy to ADHD medication in adults with ADHD, of whom 85% had a comorbid axis I or II disorder, improved ADHD symptoms and antisocial behaviour at the end of the treatment period whereas symptoms of depression, anxiety, and social functioning improved after 3 months of continued follow-up [120]. The available evidence suggests benefit of a multimodal approach in individuals with ADHD and comorbid psychiatric disorders, but large prospective studies are needed to definitively address the magnitude of benefit for ADHD and mood symptoms.

### Answers to frequently asked clinical questions

- What are the risks of prescribing psychostimulants to a patient with ADHD plus bipolar disorder or anxiety? Will it precipitate a switch to mania or an exacerbation of anxiety?

A review of previous studies supports stimulants as a first-line therapy for the treatment of ADHD symptoms in individuals with concurrent ADHD and bipolar disorder, given a lack of strong evidence that stimulants are linked to mania [17]. However, there is a theoretical risk of stimulant therapy resulting in mood destabilization in individuals with bipolar disorder [16]. Therefore, the Canadian Network for Mood and Anxiety Treatments (CANMAT) recommends bupropion as first-line therapy for individuals with comorbid ADHD and bipolar disorder, followed by stimulants. In individuals with anxiety, mixed amphetamine salts were found to be well tolerated as an adjunctive treatment to SSRIs, SNRIs, or other antidepressants [114].

b) *What should I treat first: the ADHD or the comorbid psychiatric disorder?*

**Table 3:** Summary of CADDRA guidelines for treatment of ADHD and comorbid psychiatric disorders [107]

Psychiatric comorbidity	Treatment priority
Bipolar disorder	Treat bipolar disorder first Treat ADHD once bipolar disorder is stabilized Refer to specialist
Depression Mild or dysthymia	Treat the most impairing condition first Consider treating ADHD first Consider cognitive behavioural therapy (CBT)
Moderate or severe	Treat depression first and assess suicide risk Stimulants can be combined with most antidepressants with monitoring Consider CBT
Anxiety disorders	Treat the most impairing disorder first “Start low, go slow” but titrate up to a therapeutic dose Consider adjunctive CBT Refer to specialist for augmentation with stimulants
SUD	Treat SUD first using multimodal interventions including CBT and self-help groups Treat ADHD once SUD stabilized Some cases may require concurrent treatment of SUD and ADHD
Personality disorders Borderline	Treating ADHD may facilitate psychological treatments for borderline personality disorder
Antisocial	Complex, individualized and comprehensive intervention is recommended

The consensus in the literature is that the most severe, functionally impairing or destabilizing illness should be treated first, and comorbidities should be addressed in a stepwise fashion once the patient has responded to treatment [17, 69, 107]. Generally, in patients with comorbid ADHD and mood disorders, the affective disorder should be given higher priority, and residual ADHD symptoms can then be assessed once the mood disorder has been addressed pharmacologically [84]. In bipolar disorder, mood stabilization is a prerequisite for effective ADHD treatment [76]. Similarly, in SUD it is generally recommended that substance abuse should be stabilized first [121]. The CADDRA (2011) guideline recommendations for treating ADHD and comorbid psychiatric disorders are summarized in Table 3.

c) *What should I do if my patient is smoking marijuana, or has a history of cocaine use?*

Patients with ADHD and SUD require multimodal treatment, and ADHD treatment should be delayed until the SUD has been addressed [107]. Marijuana use may have long-term negative consequences on attention, and therefore the CADDRA guidelines do not support the treatment of ADHD symptoms while a patient is taking marijuana for self-medication or recreationally [107].

Although there have been some concerns that stimulant use in the treatment of ADHD may cause increased cocaine cravings or use, this was not supported in a study of methylphenidate [121]. If diversion or misuse of medication is a concern, studies have found that extended-release stimulants are not diverted or misused in the same manner as immediate-release stimulants [122]. For instance, Levin et al. (2015) concluded that extended-release mixed amphetamine salts resulted in a reduction in cocaine use, and can be safely prescribed to patients with a history of cocaine use [123]. Another alternative to stimulants is bupropion, which has a lower abuse potential, and has effectively been used in patients with ADHD, cocaine use, and comorbid depression [132]. Overall, where abuse is a concern, non-stimulants or extended-release stimulants are preferable to immediate-release stimulants [97].

Marijuana abuse has been associated with deficits in cognition [124, 125]. However, recent research has explored the effect of cannabidiol, a cannabinoid with potential neuroprotective effects in the CNS [126], which may play a role in counteracting the dysfunction caused by tetrahydrocannabinol (THC), the high-inducing cannabinoid [127–129], and may be a precognitive factor [130, 131]. Much more investigation is required to examine the potential costs and benefits of cannabidiol.

d) *Will treating ADHD improve outcomes in depression, bipolar disorder, or SUD?*

Effective treatment of ADHD has been shown to improve comorbid disorders including SUD [69, 95, 132], bipolar disorder [17], depression [69], and anxiety disorders [114, 133]. One study hypothesized that ADHD treatment with methylphenidate or bupropion reduced cocaine use because it facilitated successful utilization of non-pharmacological interventions, or it addressed underlying deficits in dopamine function [132]. It has been hypothesized that the improvement in ADHD symptoms following treatment results in decreased functional impairment and increased quality of life, thereby reducing symptoms of comorbid anxiety or depression [114, 134]. However, it has also been noted that ADHD treatment may not be as effective in individuals with active depression [16]. The CANMAT guidelines for comorbid ADHD and MDD are summarized in Table 4.

**Table 4:** Summary of CANMAT guidelines for the management of ADHD and MDD [16]

Line of therapy	Recommended treatment(s)
First-line	Bupropion Antidepressant + long-acting stimulant
Second-line	Desipramine Nortriptyline Venlafaxine
Third-line	Antidepressant + short-acting stimulant Antidepressant + atomoxetine Antidepressant + lisdexamfetamine

Finally, it should be reiterated that the best practice for comorbid ADHD and psychiatric disorders is the principle of treating the most serious or debilitating condition first and proceeding with treatment of subsequent residual symptoms in a stepwise manner [69]. If depression is the most functionally disabling condition, its effective treatment may improve symptoms of frustration, anxiety, irritability, and concentration [69]. If ADHD is the most disabling condition in the presence of mild depression, a long-acting psychostimulant may improve ADHD symptoms and also the resultant demoralization, distress and mild depressive symptoms [69]. If both conditions are equally debilitating, treatment for both conditions can be initiated in close succession, but preferably not at exactly the same time, so that side effects or lack of efficacy can be attributed to a single intervention [69].

e) *Why have psychostimulants failed in trials of treatment-resistant depression?*

Psychostimulants have been evaluated in clinical trials for the treatment of resistant forms of depression. Although rapid antidepressant effects have been observed, such effects tend to be transient [135]. One randomized, double-blind, placebo-controlled trial of augmentation with methylphenidate in individuals with treatment-resistant depression did not find a statistically significant improvement in individuals receiving the stimulant versus placebo [136]. There has been equivocal evidence for the efficacy of lisdexamfetamine dimesylate augmentation therapy in adults with MDD, with phase 2 studies reporting positive effects on residual depressive symptoms [137] and/or symptoms of executive dysfunction [138] but this was not replicated in phase 3 studies [139]. Richards et al. (2016) propose that “the lack of efficacy observed in [the phase 3 studies] may indicate that psychostimulants as a class are ineffective in treating undifferentiated residual depressive symptoms in individuals who exhibit an inadequate response to antidepressant monotherapy” [139]. Overall, studies do not support the use of stimulants for managing treatment-resistant depression, though they may be helpful for treating fatigue and somnolence [140].

f) *Can early treatment of ADHD prevent the onset and/or mitigate the severity of depression, bipolar disorder, or SUD?*

There is some evidence to suggest that early treatment of ADHD can have a protective effect on future development of depression, bipolar disorder, and SUDs. Halmøy et al. (2009) reported that individuals with ADHD who received stimulant treatment in childhood had lower rates of depression and bipolar disorder later in life compared with individuals who were not treated [141]. A similar trend has also been observed with substance abuse [8, 142]. Two reviews of the literature concluded that stimulant therapy in children with ADHD is associated with a decreased risk of developing SUDs in the future of approximately 50% [142, 143]. Although the precise mechanism of this protective effect is not currently known, one hypothesis is that the reduction in symptoms of ADHD following treatment leads to improved academic and occupational success, and higher self-esteem, thereby reducing the risk of later substance use [143].

Early ADHD treatment can also have beneficial effects that extend beyond comorbidities. For instance, Halmøy et al. (2009) reported that children who received ADHD treatment had rates of unemployment three times lower than individuals who did not receive treatment for their ADHD as children [141].

g) *Are psychostimulants addictive?*

Psychostimulants such as methylphenidate and mixed amphetamine salts have been demonstrated to possess likeability and euphorogenic effects, and are therefore classified as Schedule II controlled substances with abuse liability potential [144]. Despite this potential, there is extremely limited evidence in the literature of psychostimulant abuse among ADHD patients [145, 146]. The European Consensus Statement on Diagnosis and Treatment of Adult ADHD [71] concludes that psychostimulants are not addictive, as there is no evidence of tolerance over time, and non-compliance or cessation is a risk with stimulant medications, rather than overuse. However, where patients are at higher risk of drug misuse or diversion, longer-acting stimulant formulations or non-stimulants should, in most cases, be used.

h) *Are psychostimulants overprescribed and overused in college/university students?*

There is some concern that college or university students may exaggerate ADHD symptoms in order to obtain a stimulant prescription, which they perceive as helpful in terms of academic performance [147]. There is also some evidence for issues with stimulant diversion and misuse among college and university students. Misuse of prescription stimulants has been reported in approximately 7% of college students, both to improve concentration and academic performance, as well as for recreational purposes [32]. Recent research supports that most non-prescription use of psychostimulants among college and university students is to aid in concentration, and often where students report attentional difficulties that are hindering academic success [144, 148]. Students may thus be self-medicating in order to treat undiagnosed ADHD. This highlights the importance of diagnosing college and university students with ADHD in order to provide them with supervised pharmacotherapy where needed, as well as educating individuals with psychostimulant prescriptions about the consequences of diverting their medication.

## Conclusion

ADHD is a prevalent psychiatric disorder in the adult population that is frequently unrecognized, under-diagnosed, and under-treated. Given that it is often comorbid with other psychopathologies including mood or anxiety disorders, substance use disorders, and personality disorders, adults presenting with symptoms of ADHD should be screened for these frequently comorbid conditions, and vice versa, in order to identify patients who could potentially benefit from optimal management of ADHD and its comorbidities. Although the clinical presentation of ADHD in adults can be variable and complex, it can often be identified using a few high-yield clinical questions, and the use of validated assessment scales in patients screening positive. Early and optimal treatment of ADHD has the potential to change the trajectory of psychiatric morbidity later in life and to substantially improve functional outcomes across the spectrum of psychiatric comorbidities. In general, when ADHD coexists with other psychiatric pathologies, the more severe disorder should be treated first according to evidence-based guidelines. In the coming years, research on the genetic and neurobiological basis of ADHD should continue to uncover fruitful avenues of treatment for this debilitating disorder and ultimately improve outcomes for patients and their families.

## Abbreviations

AAQoL: Adult ADHD Quality of Life Scale
ACC: anterior cingulate cortex
ADHD: attention-deficit/hyperactivity disorder
ASRS: Adult ADHD Self-Report Scale
CAARS: Conners’ Adult ADHD Rating Scales
CADDRA: Canadian ADHD Resource Alliance
CANMAT: Canadian Network for Mood and Anxiety Treatments
CBT: cognitive behavioural therapy
CI: confidence interval
DA: dopamine
dlPFC: dorsolateral prefrontal cortex
DSM-5: Diagnostic and Statistical Manual of Mental Disorders, 5th Edition
DSM-IV-TR: Diagnostic and Statistical Manual of Mental Disorders, 4th Edition Text Revision
FAST MINDS: Forgetful; Achieving below potential; Stuck in a rut; Time challenged; Motivationally challenged; Impulsive; Novelty seeking; Distractible; Scattered
GABA: gamma-aminobutyric acid
HCl: hydrochloride
IQ: intelligence quotient
LCSPT: limbic-cortical-striatal-pallidal-thalamic
MDD: major depressive disorder
NE: norepinephrine
OMPFC: orbital and medial prefrontal cortex
PFC: prefrontal cortex
RDoC: Research domain criteria
SNRI: serotonin-norepinephrine reuptake inhibitor
SR: sustained release
SSRI: selective serotonin reuptake inhibitor
SUD: substance use disorder
THC: tetrahydrocannabinol
TRD: treatment resistant depression
WFIRS: Weiss Functional Impairment Rating Scale
WRAADDS: Wender-Reimherr Adult Attention-Deficit Disorder Scale
XR: extended release
